# Access to primary care and the route of emergency admission to hospital: retrospective analysis of national hospital administrative data

**DOI:** 10.1136/bmjqs-2015-004338

**Published:** 2015-08-25

**Authors:** Thomas E Cowling, Matthew Harris, Hilary Watt, Michael Soljak, Emma Richards, Elinor Gunning, Alex Bottle, James Macinko, Azeem Majeed

**Affiliations:** 1 Department of Primary Care and Public Health, Imperial College London, London, UK; 2 Department of Nutrition, Food Studies, and Public Health, New York University, New York, New York, USA

**Keywords:** Health services research, Primary care, Ambulatory care, Emergency department, General practice

## Abstract

**Background:**

The UK government is pursuing policies to improve primary care access, as many patients visit accident and emergency (A and E) departments after being unable to get suitable general practice appointments. Direct admission to hospital via a general practitioner (GP) averts A and E use, and may reduce total hospital costs. It could also enhance the continuity of information between GPs and hospital doctors, possibly improving healthcare outcomes.

**Objective:**

To determine whether primary care access is associated with the route of emergency admission—via a GP versus via an A and E department.

**Methods:**

Retrospective analysis of national administrative data from English hospitals for 2011–2012. Adults admitted in an emergency (unscheduled) for ≥1 night via a GP or an A and E department formed the study population. The measure of primary care access—the percentage of patients able to get a general practice appointment on their last attempt—was derived from a large, nationally representative patient survey. Multilevel logistic regression was used to estimate associations, adjusting for patient and admission characteristics.

**Results:**

The analysis included 2 322 112 emergency admissions (81.9% via an A and E department). With a 5 unit increase in the percentage of patients able to get a general practice appointment on their last attempt, the adjusted odds of GP admission (vs A and E admission) was estimated to increase by 15% (OR 1.15, 95% CI 1.12 to 1.17). The probability of GP admission if ≥95% of appointment attempts were successful in each general practice was estimated to be 19.6%. This probability reduced to 13.6% when <80% of appointment attempts were successful. This equates to 139 673 fewer GP admissions (456 232 vs 316 559) assuming no change in the total number of admissions. Associations were consistent in direction across geographical regions of England.

**Conclusions:**

Among hospital inpatients admitted as an emergency, patients registered to more accessible general practices were more likely to have been admitted via a GP (vs an A and E department). This furthers evidence suggesting that access to general practice is related to use of emergency hospital services in England. The relative merits of the two admission routes remain unclear.

## Introduction

Access to primary care and its impact on demand for emergency hospital care is a topical issue in many developed countries.^1^ In the UK, the government is piloting a scheme whereby primary care practices (general practices) offer appointments from 08:00 to 20:00, 7 days a week and use teleconsultations more widely.^2^ This policy, initially trialled in approximately 14% of general practices in England, is now planned to be implemented nationally.^3^ The government expects that increasing access to general practice will reduce demand for accident and emergency (A and E) services.^4^

English general practices have registered lists of patients (mean=7267)^5^ for whom they provide a comprehensive range of services and a first point of contact within the National Health Service (NHS). Patients typically access care by appointment between 08:00 and 18:30, Monday to Friday. Outside of these times, access to primary care varies widely across the country, but may include designated telephone services, out-of-hours clinics and home visits.^6^ A and E services range from consultant-led emergency departments (open all day) to general practitioner (GP) or nurse-led services intended to treat minor illness (varied opening times), which are accessible without appointment. Other sources of urgent care include a national telephone helpline, pharmacists and ambulance services.^7^

The annual number of visits to A and E departments in England increased from 18.8 million in 2005–2006 to 22.4 million in 2014–2015,^8^ amid reports of these services being stretched by demand. An estimated 26.5% of these visits follow unsuccessful attempts to get suitable general practice appointments.^9^ Studies often examine this mechanism for patients who could be managed in a primary care setting, outside of an A and E department,^10^ ^11^ but it may also apply to patients who are ultimately admitted to hospital, such that access to general practice could be associated with the route of admission. No studies have yet investigated this association.

Direct admission via a GP, instead of via an A and E department, averts A and E use, and may reduce total hospital costs. It may also improve communication and coordination of care between primary care and hospital services, given that a GP is in early contact with hospital staff. This could lead to enhanced continuity of information or patient management between GPs and hospital doctors, possibly contributing to shorter lengths of stay and better follow-up after discharge. The association between access to general practice and the route of admission is also of scientific interest in itself, in further characterising the relationship between features of primary care and the use of unscheduled hospital care.

We hypothesised that patients who have an emergency (unscheduled) admission are more likely to be admitted via a GP (vs an A and E department) if they are registered to a more accessible general practice. We also expected to observe this association for three specific conditions (chronic obstructive pulmonary disease, pneumonia and urinary tract infections), but not for a ‘control’ condition (fractures of the lower limb), in subgroup analyses. To investigate this, we conducted a patient level analysis of national hospital administrative data linked with a large, nationally representative patient survey in England.

## Methods

### Study design, setting and main data source

We conducted a retrospective analysis of admitted patient care records from the Hospital Episode Statistics database. The study period was from 1 April 2011 to 31 March 2012.

The initial dataset included all emergency admissions to English NHS hospitals during the study period. Each record in the data corresponded to a finished consultant episode, a continuous period of time for which a patient was under the care of the same consultant. We linked episodes to form admissions—continuous periods of care to final discharge after any transfers (within or between hospitals)—and used these as the unit of analysis.^12^ ^13^

### Study population

The analysis included emergency admissions to all (n=147) non-specialist acute hospital providers^14^ for patients aged ≥18 years old who were resident in and registered to a general practice in England.

Admissions where the patient was admitted and discharged (alive) on the same calendar day were excluded, as some of these likely related to the activity of medical assessment/clinical decision units rather than inpatient wards.^15^ Records with invalid or missing values for age, sex, route of admission, method of discharge, primary diagnosis, lower layer super output area (LSOA) of residence or general practice code were also excluded (2% of remaining admissions). We categorised primary diagnoses, coded using the International Statistical Classification of Diseases and Related Health Problems 10th Revision,^16^ into Clinical Classification Software (CCS) groups,^17^ which are often used for the analysis of admitted patient care in England and elsewhere.^14^ ^18^ CCS groups relating to mental health, injury and poisoning were excluded from the analysis, as the relevance of the hypothesis to these groups was less clear (see online supplementary appendix 1).

### Outcome variable

Emergency admissions are defined in the English NHS as admissions that are unpredictable and occur at short notice because of clinical need.^19^ The route of emergency admission was classed as ‘via an A&E department’ if ‘Emergency: via Accident and Emergency (A&E) services, including the casualty department of the provider’ or ‘Emergency: other means, including patients who arrive via the A&E department of another healthcare provider’ was recorded. Admissions were categorised as ‘via a GP’ if ‘Emergency: via general practitioner’ was recorded; these admissions are defined as those occurring after a request for immediate admission has been made direct to a hospital by a GP or deputy.^19^

We excluded emergency admissions via a Bed Bureau or consultant outpatient clinic (<5% of admissions), as we were primarily interested in the two routes accounting for the vast majority of admissions, the results were simpler to present with a binary outcome, and we expected the relative odds between GP and A and E admissions to be largely independent of other admission routes.

### Explanatory variables

We derived the measure of access to general practice from the GP Patient Survey 2011–2012.^20^ All general practices in England with eligible patients (aged ≥18 years old, general practice registered and with a valid NHS number) were included in the survey (n=8271).^20^ Questionnaires were delivered to 2 624 585 (alive) patients (approximately 5% of the national population^21^), with an overall response rate of 40% (n=1 037 946).^20^ The median number of responses and response rate per practice were 128 (IQR 113–142) and 40% (IQR 32–48%), respectively.

Measures of access previously derived from GP Patient Survey questions have demonstrated high practice-level reliability (ratio of the between-practice variance of practice-level means to the total variance at the observed sample size exceeds 0.9).^22^ The same measures are largely unassociated with rates of non-response once population characteristics are controlled for (response rate accounts for less than 0.2% of variation in measures) and have shown construct validity.^23^ ^24^ A weighting scheme was applied to the results to account for sampling design and differential response patterns.^20^ Questions are tested in cognitive interviews before use.^20^

We use the term ‘access’ to refer to patients’ abilities to receive healthcare, which here we operationalise as the ability to get an appointment. For each general practice, we calculated the weighted percentage of respondents that had been able to obtain an appointment to see or speak to a GP or nurse from their general practice on their last attempt.^9^ Each patient admitted to hospital was assigned the percentage calculated for the general practice to which they were registered. A small number of admissions (0.4%) were excluded due to missing data for the access measure.

Ethnicity was ‘white’, ‘mixed’, ‘Asian’, ‘black’, ‘other’ or ‘not known’ (5% of admissions) as recorded in Hospital Episode Statistics data. We assigned each patient a socioeconomic status score equal to the national rank of the Index of Multiple Deprivation^25^ value for the LSOA (small geographical area with a mean population of 1500) in which the patient resided. This LSOA also indicated the urban/rural setting and English region of each patient's residence.^26^ A modified Charlson index, using weights adapted for England, was the comorbidity measure.^18^ ^27^ We calculated the risk of GP admission for each CCS diagnosis group as the percentage of admissions that were via a GP for that group. Some diagnoses are more frequent in GP admissions than A and E admissions, and these account for greater percentages of admissions for some populations, perhaps due to disease prevalence. These same areas may incidentally have greater access to general practice. The diagnosis risk variable, therefore, provided additional adjustment for the case mix of patients.^13^

### Statistical methods

We estimated the associations between the log odds of admission via a GP and age, sex, ethnicity, socioeconomic status, comorbidity, urban/rural area of residence, diagnosis risk of GP admission, access to general practice, the day and month of admission and the region of residence, using logistic regression. We coded age and socioeconomic status as categorical variables to account for the non-linear associations observed. The comorbidity variable was excluded from the multivariable model as its correlation with the access measure was very weak (r=0.005), and it did not show a linear or polynomial relationship with the outcome variable; comorbidity was not a confounding variable.

In the multivariable analysis, we fitted a multilevel model with a random intercept for each general practice to account for the clustering of patients within practices. Access to general practice was coded both as a continuous and as a categorical variable in separate models to facilitate interpretation of the association with the outcome variable. The limits of the categorical variable, where x is the percentage of appointment attempts that were successful, were: x<80, 80≤x<85, 85≤x<90, 90≤x<95, 95≤x≤100; these were chosen to provide five groups with similar ranges across the distribution of access to general practice.

In our final models, we included interactions between access to general practice and urban/rural area and region of residence, to test whether any association between the route of admission and access to general practice varied by area. Such differences could reflect geographical variation in service design or healthcare seeking behaviour. All multivariable models for the above pooled analysis across conditions were estimated using a random 50% sample of admissions, to shorten computation time.

We repeated the methods of the pooled analysis for three specific conditions specified a priori: chronic obstructive pulmonary disease (CCS 127), pneumonia (CCS 122) and urinary tract infections (CCS 159). Hospital admissions for these conditions are often considered to be particularly sensitive to primary care factors.^28^ They are also among the five most frequently recorded for both GP and A and E admissions. To assess residual confounding, we also analysed admissions for fractures of the lower limb (CCS 230), which we had previously excluded as our hypothesis was not applicable to it.

The results of the logistic regression models are presented as ORs and margins of responses. The margins here are the average predicted probabilities of admission via a GP when all patients were given a particular value of access to general practice, with all other explanatory variables retaining the actual values observed in the dataset.^29^ These margins allow results to be interpreted in terms of adjusted probabilities, rather than adjusted odds. To estimate the corresponding absolute effects in the total population, we multiplied the margins of responses by the number of admissions. Data processing and analysis were conducted using Stata MP V.13.1 (Stata, College Station, Texas, USA).

## Results

The analysis included 2 322 112 emergency hospital admissions in England in 2011–2012. Most patients, 81.9% (1 902 864), were admitted via an A and E department. The median percentage of patients able to obtain a general practice appointment on their last attempt was 91.6% (IQR 87.5%–94.6%, range 59.8%–100%) (table 1).

**Table 1:** Descriptive statistics of study population, by route of admission

	A and E admission (n=1 902 864)	GP admission (n=419 248)
Age (years)	69 (50 to 81)	68 (48 to 80)
Female (%)	51.5	55.1
Ethnicity (%):		
White	86.1	90.3
Mixed	0.5	0.3
Asian	4.7	2.4
Black	2.6	0.8
Other	1.6	0.6
Not known	4.5	5.7
Index of Multiple Deprivation rank*	43 (20 to 69)	49 (25 to 73)
Charlson index of comorbidity	3 (0 to 11)	3 (0 to 10)
Urban area of residence (%)	83.9	75.8
Diagnosis risk of GP admission†	18.3 (13.3 to 22.8)	21.7 (15.8 to 25.9)
Access to general practice‡	91.4 (87.3 to 94.5)	92.4 (88.8 to 95.2)
Access to general practice (x) (%)‡		
* *x<80 (least accessible)	5.0	2.7
* *80≤x<85	11.4	8.4
* *85≤x<90	23.8	21.3
90≤x<95	38.2	40.6
* *95≤x≤100 (most accessible)	21.6	27.0

Region of residence (percentage of total): East Midlands (8.5%), East of England (10.4%), London (13.0%), North East (5.8%), North West (15.5%), South East (14.7%), South West (9.9%), West Midlands (11.0%), Yorkshire and the Humber (11.3%).

Statistics given as median (IQR) for continuous variables and as column percentages for categorical variables.

*Centiles of rank; greater centiles correspond to lower Index of Multiple Deprivation scores (less deprivation).

†The percentage of admissions for a given Clinical Classification Software group that were via a GP.

‡Percentage of GP Patient Survey respondents registered to the patient's general practice who were able to obtain a general practice appointment on their last attempt.

A and E, accident and emergency; GP, general practitioner.

Half of admissions (50.2%) were for patients aged ≥69 years old. Pneumonia was the most frequent primary diagnosis (133 287 admissions, 5.7%), while urinary tract infections (110 078, 4.7%) and chronic obstructive pulmonary disease (97 529, 4.2%) were the third and fifth most common, respectively (see online supplementary appendix 2).

Patients registered to more accessible general practices were more likely to be admitted via a GP (vs an A and E department) than those registered to less accessible practices (for a 5 unit increase in the percentage of patients able to obtain an appointment, OR of GP admission is 1.21, 95% CI 1.21 to 1.22). The percentages admitted via a GP in the five categories of access to general practice, from the least to the most accessible practices, were 10.7%, 14.0%, 16.5%, 19.0% and 21.6%.

**Figure 1:**
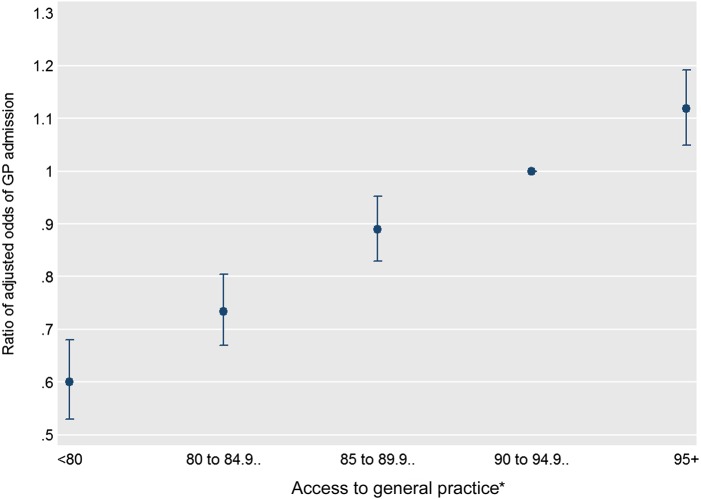
Adjusted ORs of general practitioner (GP) admission (vs A and E admission) and 95% CIs, by access to general practice, derived from a multivariable multilevel logistic regression model (pooled analysis). *Percentage of GP Patient Survey respondents registered to the patient's general practice who were able to obtain a general practice appointment on their last attempt.

In the multivariable analysis, the ratios of adjusted odds of GP admission across the five access categories, from the least to the most accessible practices, were 0.60 (95% CI 0.53 to 0.68), 0.73 (0.67 to 0.80), 0.89 (0.83 to 0.95), 1 (reference) and 1.12 (1.05 to 1.19) (figure 1 and see online supplementary appendix 3). For a 5 unit increase in the measure of access, the adjusted odds of GP admission was estimated to increase by 15% (OR 1.15, 95% CI 1.12 to 1.17).

The association was slightly greater in rural areas (for a 5 unit increase in access, rural OR 1.17, urban OR 1.15, p<0.05 for interaction). It also varied by English region (p<0.01 for joint test of all region interactions; see online supplementary appendix 4). Patients were more likely to be admitted via a GP if registered to a more accessible general practice in all nine regions; the association was not statistically significant at the 5% level in the North East only (OR 1.01, p=0.47).

In the final model (including interaction terms for both urban/rural area and region of residence), the average probability of GP admission if all practices had a value from 95% to 100% for the access measure was estimated to be 19.6%. If all practices had a value less than 80%, the average probability was 13.6%. This decrease in the probability equates to 139 673 fewer GP admissions (456 232 vs 316 559) assuming no change in the total number of admissions (via either route) (table 2).

**Table 2:** Predicted probabilities of GP admission (vs A and E admission) and corresponding expected numbers of GP admissions, according to access to general practice, derived from multivariable multilevel logistic regression models

	Predicted probabilities of GP admission, by access to general practice*					Expected numbers of GP admissions, by access to general practice*†				
	60	<80	Actual	≥95	100	60	<80	Actual	≥95	100
All conditions	10.0	13.6	17.9	19.6	20.9	231 183	316 559	414 842	456 232	484 773
Subgroups										
COPD	8.7	11.0	14.7	16.2	17.0	8 532	10 758	14 341	15 763	16 576
Pneumonia	7.7	10.8	14.9	16.6	17.6	10 285	14 457	19 826	22 075	23 437
Urinary tract infections	10.9	12.5	17.4	18.5	19.7	12 025	13 766	19 109	20 390	21 690

Results adjusted for age, sex, ethnicity, socioeconomic status, urban/rural area of residence, diagnosis risk of general practice admission, the day and month of admission and the region of residence.

60, minimum value of access; <80, lowest access category; ≥95, highest access category; 100, maximum value of access; actual, access as recorded in dataset.

*Percentage of GP Patient Survey respondents registered to the patient's general practice who were able to obtain a general practice appointment on their last attempt.

†Obtained by multiplying the predicted probabilities by the number of admissions in the sample (all conditions: 2 322 112).

A and E, accident and emergency; COPD, chronic obstructive pulmonary disease; GP, general practitioner.

Of the total residual variance in individuals’ propensities to be admitted via a GP (vs via A and E), 26% was due to unobserved differences between practices (with the remaining 74% due to residual variance between individuals) (based on the variance partition coefficient of 0.26 in the final model).

**Figure 2:**
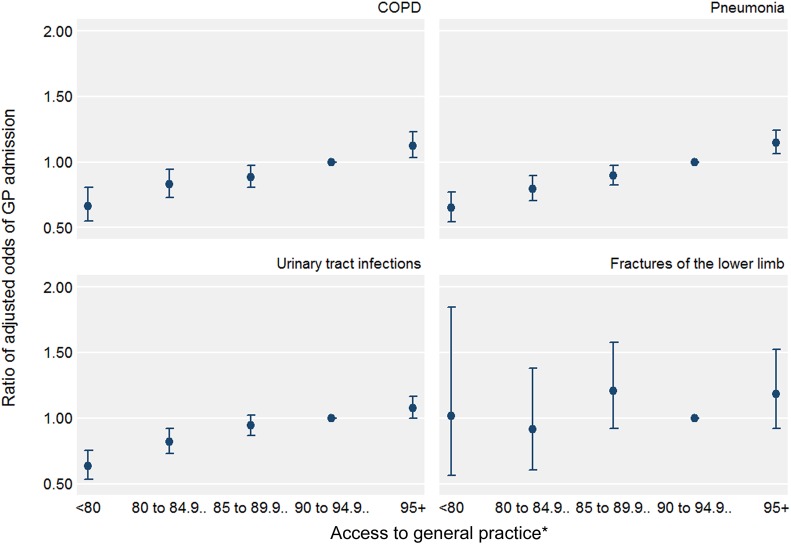
Adjusted ORs of GP admission (vs A and E admission) and 95% CIs, by access to general practice, derived from multivariable multilevel logistic regression models (subgroup analysis). *Percentage of GP Patient Survey respondents registered to the patient's general practice who were able to obtain a general practice appointment on their last attempt. COPD, chronic obstructive pulmonary disease; GP, general practitioner.

The association estimated in the pooled analysis across conditions was similar to those estimated in the subgroup analyses of chronic obstructive pulmonary disease, pneumonia and urinary tract infections (table 2 and figure 2). For example, for pneumonia, the adjusted ORs across the five access categories were 0.65 (95% CI 0.55 to 0.77), 0.80 (0.71 to 0.90), 0.90 (0.82 to 0.98), 1 (reference) and 1.15 (1.06 to 1.24).

For fractures of the lower limb (the ‘control’ condition) the estimated association between access to general practice and the odds of GP admission was not statistically significant (for a 5 unit increase, OR 1.05, 95% CI 0.94 to 1.16, p=0.38; figure 2 shows the absence of a linear trend in the odds).

## Discussion

### Summary of findings

Among patients who had an emergency hospital admission, those registered to more accessible general practices were more likely to have been admitted via a GP (vs an A and E department) than those registered to less accessible practices. This association showed a clear gradient, and was highly statistically significant before and after adjusting for patient and admission characteristics. It was consistent in direction between geographical regions of England. The findings for chronic obstructive pulmonary disease, pneumonia and urinary tract infections were consistent with the pooled analysis of all conditions examined.

### Strengths and limitations

The analysis used a national hospital dataset linked with other established data sources to address a prominent topic in contemporary health policy in several countries. To our knowledge, it is the first national, patient level analysis of the association between access to primary care and the route of emergency admission.

One limitation is that the recorded route of admission may not entirely reflect a patient's pathway to admission, given, for example, the presence of medical observation units located alongside A and E departments to which GPs can directly refer patients in many hospitals. These instances could have biased the association towards the null, and therefore, the unbiased associations may be larger than observed. To address the possibility that service (or coding) differences between English regions influence the results (such as few GP admissions in London), we included interaction terms for each region in our models. The completeness of the route of admission field in Hospital Episode Statistics is satisfactory, with 0.05% of episodes missing data. We are unaware of any attempts to validate the recorded route of admission with primary data.

We assumed that patients registered to the same general practice have the same access to appointments, since we assigned a practice-level measure to individual patients. While certain types of patients tend to report worse experiences within practices (such as younger patients),^30^ adjustment of practice-level means for characteristics of responders has only a small effect on measures of access (≤2.0% of variation in practice-level means due to case-mix adjustment).^31^

Our analysis examined the association between access to primary care and the setting of acute care in the context of emergency admissions only, such that the findings are not necessarily generalisable to all acute care. In this context, with greater levels of illness, patients may be more inclined to visit an A and E department if they are unable to obtain a suitable general practice appointment. Conversely, these patients may be more likely to visit an A and E department without considering access to primary care beforehand.

### Relation to existing literature

The percentage of emergency admissions in England that are A and E admissions increased year on year from 2001–2002 to 2010–2011, driven by a large increase in numbers of A and E admissions and a reduction in GP admissions.^32^ Similar trends exist in the USA.^33^

In the US Nationwide Inpatient Sample 2000–2009, patients were more likely to be admitted via an A and E department (vs a physician office) if they were elderly, non-white or were admitted at the weekend;^33^ these findings are similar to those reported here, but the US analysis did not examine associations with access to primary care. In a sample of adults from one Canadian region, respondents were more likely to report that their last GP contact was in an A and E department (vs all other locations) if they did not have a regular or family physician;^34^ this analysis excluded respondents who reported a hospital admission in the previous 12 months.

In England, patients are more likely to report using out-of-hours primary care services if they have worse in-hours access to their general practice, according to the GP Patient Survey.^35^ Several studies have examined the cross-sectional, ecological association between access to primary care and rates of A and E visits in England;^11^ ^36^ ^37^ the sole national analysis observed that more accessible general practices have lower rates of A and E visits.^11^ In the USA and Canada, similar findings have been reported from national patient surveys^38–40^ or local studies using administrative data.^41^ ^42^

### Potential explanations for findings

Our findings of increased odds of GP admission with increasing access to general practice could be explained in part by some patients, who require hospital admission, visiting an A and E department after perceiving or experiencing difficulties in obtaining a suitable general practice appointment. An A and E admission may occur ‘instead of’ a GP admission. An exploratory analysis of the GP Patient Survey estimated that approximately 26.5% (5.77 million) of A and E visits in England in 2012–2013 were preceded by patients being unable to obtain a convenient general practice appointment.^9^ Under this hypothesis alone, access to primary care affects the percentage of admissions that are via a given route, but it does not affect the total number or rate of admissions.

A further hypothesis is that greater access to primary care can help prevent some admissions from occurring at all. Several national studies have estimated that more accessible general practices in England have lower adjusted rates of emergency admissions for chronic obstructive pulmonary disease,^43^ diabetes complications^44^ and others,^45–48^ and lower odds of emergency admission (vs elective admission) for cancer.^49^ The presented estimates of the expected number of GP admissions with variation in access to general practice, which assume the total number of admissions remains constant, should, therefore, be used to help interpret the probabilities only. Both of the above explanations for the findings—redistribution of patients to favour GP admissions and reductions in the total number of admissions with greater access to general practice—are plausible.

### Implications for policy and research

The relative merits of admission via a GP and via an A and E department are currently unclear. We have outlined several potential benefits of direct admission via a GP above, including improved information continuity between GPs and hospital doctors. GPs may also have a higher threshold for admitting patients than emergency department doctors, thereby possibly preventing less serious cases from being needlessly admitted. However, GP admission could be associated with a longer time to admission, partly due to delays in getting an appointment, with adverse effects on patient health outcomes and satisfaction.^33^ ^50^ Future research could examine the advantages and disadvantages of different admission routes.

The generalisability of our findings to other countries is unclear, particularly to those with different arrangements for the delivery and financing of health services. Future research could, therefore, suitably repeat the analysis in other contexts and examine the effects of relevant natural experiments in each, such as the increase in health insurance coverage in the USA^51^ ^52^ and the extension of primary care practice opening hours in Italy.^53^

Our results provide further evidence to suggest that variation in access to general practice is related to usage of emergency hospital services in England. This lends support to, but does not fully endorse, national policy expectations that practices improving access will affect use of A and E services. No studies have convincingly examined the longitudinal associations between access to primary care and use of unscheduled hospital care, and experimental evidence on interventions designed to improve access is lacking. Our findings should motivate work addressing these gaps in knowledge, particularly that relevant to general practice opening hours.^4^ The UK government should pause its planned extension of opening hours nationally until a sufficient evidence base has been established.

## Supplementary Material

Web supplement
