# Supplementary material for: Access to primary care and the route of emergency admission to hospital: retrospective analysis of national hospital administrative data
Source: BMJ Qual Saf. 2015 Aug 25;25(6):432–40. doi: 10.1136/bmjqs-2015-004338 (PMC4893129; doi:10.1136/bmjqs-2015-004338)
Supplement: Web supplement [file bmjqs-2015-004338-s1.pdf]

Appendix 1 Clinical Classification Software (CCS) groups excluded from the analysis

CCS groups relating to mental health were excluded as the potential relevance of access to primary care to the route of admission was unclear. CCS groups relating to injury and poisoning were excluded as patients are likely to visit an accident and emergency (A&E) department immediately, irrespective of access to their registered general practice.

**CCS group**	**CCS label**
	Mental health
65	Mental retardation
66	Alcohol-related mental disorders
67	Substance-related mental disorders
68	Senility and organic mental disorders
69	Affective disorders
70	Schizophrenia and related disorders
71	Other psychoses
72	Anxiety; somatoform; dissociative; and personality disorders
73	Preadult disorders
74	Other mental conditions
75	Personal history of mental disorder
	Injury and poisoning
225	Joint disorders and dislocations; trauma-related
226	Fracture of neck of femur (hip)
227	Spinal cord injury
228	Skull and face fractures
229	Fracture of upper limb
230	Fracture of lower limb
231	Other fractures
232	Sprains and strains
233	Intracranial injury
234	Crushing injury or internal injury
235	Open wounds of head; neck; and trunk
236	Open wounds of extremities
237	Complication of device; implant; or graft
238	Complication of surgical procedures or medical care
239	Superficial injury; contusion
240	Burns
241	Poisoning by psychotropic drugs
242	Poisoning by other medications and drugs
243	Poisoning by nonmedicinal substances
244	Other injuries and conditions due to external causes
253	Allergic reactions

Appendix 2 Numbers of A&E admissions and GP admissions in the study population by Clinical Classification Software (CCS) group primary diagnosis

Rank	CCS group	CCS label	Number of A&E admissions	Percentage of A&E admissions (%)	Diagnosis risk of A&E admission (%)
1	122	Pneumonia*†	112 265	5.9	84.2
2	102	Nonspecific chest pain	104 009	5.5	91.1
3	159	Urinary tract infections†	89 947	4.7	81.7
4	127	COPD†	82 168	4.3	84.2
5	251	Abdominal pain	77 578	4.1	74.1

Rank	CCS group	CCS label	Number of GP admissions	Percentage of GP admissions (%)	Diagnosis risk of GP admission (%)
1	251	Abdominal pain	27 159	6.5	25.9
2	122	Pneumonia*†	21 022	5.0	15.8
3	159	Urinary tract infections†	20 131	4.8	18.3
4	197	Skin and subcutaneous tissue infections	15 760	3.8	25.5
5	127	COPD†	15 361	3.7	15.8

A&E: accident and emergency, COPD: chronic obstructive pulmonary disease, GP: general practitioner
Fractures of the lower limb (CCS 230): 40 359 admissions (39 960 A&E admissions, 399 GP admissions)
*Excludes pneumonia caused by tuberculosis or sexually transmitted disease
†Included in subgroup analysis

Appendix 3 Univariable and multivariable multilevel logistic regression models of associations between the odds of GP admission (versus A&E admission) and patient characteristics in England, 2011-12

	Unadjusted odds ratio (95% CI)	P value	Adjusted odds ratio (95% CI)	P value
**Age (years):**				
18 to 29	1		1	
30 to 39	0.93 (0.92 to 0.95)	<0.001	1.04 (1.02 to 1.07)	<0.01
40 to 49	0.89 (0.88 to 0.91)	<0.001	1.02 (1.00 to 1.05)	0.08
50 to 59	0.84 (0.83 to 0.86)	<0.001	1.03 (1.01 to 1.06)	<0.05
60 to 69	0.89 (0.88 to 0.91)	<0.001	1.05 (1.03 to 1.08)	<0.001
70 to 79	0.86 (0.84 to 0.87)	<0.001	1.04 (1.02 to 1.06)	<0.001
80 to 89	0.84 (0.83 to 0.85)	<0.001	1.01 (0.99 to 1.04)	0.21
≥90	0.78 (0.77 to 0.79)	<0.001	0.93 (0.91 to 0.96)	<0.001
**Female (versus male)**	1.16 (1.15 to 1.17)	<0.001	1.14 (1.13 to 1.15)	<0.001
**Ethnicity:**				
White	1		1	
Mixed	0.66 (0.62 to 0.69)	<0.001	0.97 (0.89 to 1.06)	0.51
Asian	0.48 (0.47 to 0.49)	<0.001	1.03 (1.00 to 1.07)	0.09
Black	0.28 (0.27 to 0.29)	<0.001	0.81 (0.76 to 0.86)	<0.001
Other	0.37 (0.35 to 0.38)	<0.001	0.77 (0.72 to 0.82)	<0.001
Not known	1.20 (1.19 to 1.22)	<0.001	1.28 (1.25 to 1.32)	<0.001
**Index of Multiple Deprivation rank:**				
1 to 6 497 (most deprived)	1		1	
6 498 to 12 993	1.18 (1.17 to 1.19)	<0.001	1.05 (1.03 to 1.07)	<0.001
12 994 to 19 490	1.40 (1.38 to 1.41)	<0.001	1.09 (1.07 to 1.11)	<0.001
19 491 to 25 986	1.45 (1.43 to 1.46)	<0.001	1.13 (1.10 to 1.15)	<0.001
25 987 to 32 482 (least deprived)	1.46 (1.44 to 1.47)	<0.001	1.15 (1.12 to 1.17)	<0.001
**Rural area of residence (versus urban)**	1.67 (1.65 to 1.68)	<0.001	1.06 (1.04 to 1.08)	<0.001
**Diagnosis risk of GP admission**	1.07 (1.07 to 1.07)	<0.001	1.08 (1.08 to 1.08)	<0.001
**Access to general practice (*x*)*:**				
x<80 (least accessible)	0.51 (0.50 to 0.52)	<0.001	0.60 (0.53 to 0.68)	<0.001
80≤*x*<85	0.69 (0.68 to 0.70)	<0.001	0.73 (0.67 to 0.80)	<0.001
85≤*x*<90	0.84 (0.83 to 0.85)	<0.001	0.89 (0.83 to 0.95)	<0.001
90≤*x*<95	1		1	
95≤*x*≤100 (most accessible)	1.17 (1.16 to 1.18)	<0.001	1.12 (1.05 to 1.19)	<0.001
**Day of admission:**				
Sunday	1		1	
Monday	2.12 (2.09 to 2.15)	<0.001	2.18 (2.13 to 2.23)	<0.001
Tuesday	2.08 (2.05 to 2.11)	<0.001	2.16 (2.12 to 2.21)	<0.001
Wednesday	2.11 (2.08 to 2.14)	<0.001	2.21 (2.16 to 2.26)	<0.001
Thursday	2.18 (2.15 to 2.21)	<0.001	2.30 (2.25 to 2.35)	<0.001
Friday	2.39 (2.35 to 2.42)	<0.001	2.52 (2.46 to 2.57)	<0.001
Saturday	1.10 (1.08 to 1.12)	<0.001	1.11 (1.08 to 1.14)	<0.001
**Region of residence:**				
East Midlands	1		1	
East of England	0.88 (0.86 to 0.89)	<0.001	0.69 (0.62 to 0.76)	<0.001
London	0.10 (0.09 to 0.10)	<0.001	0.09 (0.08 to 0.10)	<0.001
North East	0.94 (0.93 to 0.96)	<0.001	0.85 (0.75 to 0.96)	<0.05
North West	0.81 (0.80 to 0.82)	<0.001	0.76 (0.70 to 0.83)	<0.001
South East	0.85 (0.84 to 0.86)	<0.001	0.75 (0.69 to 0.82)	<0.001
South West	1.45 (1.43 to 1.47)	<0.001	1.34 (1.21 to 1.48)	<0.001
West Midlands	0.78 (0.77 to 0.79)	<0.001	0.78 (0.71 to 0.86)	<0.001
Yorkshire and the Humber	0.87 (0.86 to 0.88)	<0.001	0.89 (0.81 to 0.98)	<0.05

A&E: accident and emergency, GP: general practitioner

Multivariable model also adjusted for month of admission: January (reference), February (odds ratio 0.97), March (0.98), April (1.05), May (1.10), June (1.12), July (1.12), August (1.11), September (1.09), October (1.07), November (1.05), December (0.97); P<0.001.

*Percentage of GP Patient Survey respondents registered to the patient's general practice who were able to obtain a general practice appointment on their last attempt

Appendix 4 Predicted probabilities of GP admission (versus A&E admission), by access to general practice and region of England, derived from a multivariable multilevel logistic regression model

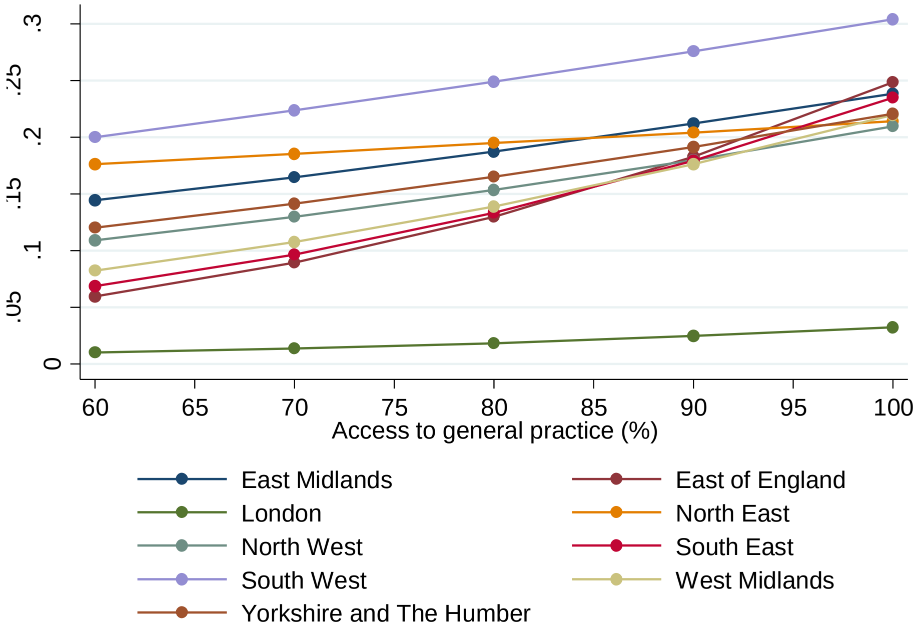

*Percentage of GP Patient Survey respondents registered to the patient's general practice who were able to obtain a general practice appointment on their last attempt

The low probability of GP admission in London could be explained in part by differences in the configuration of urgent and emergency care services, patients' healthcare seeking behaviour, data coding, and the referral behaviours/pathways of GPs.

Supporting data:

	Access to general practice (%)				
**Region**	**60**	**70**	**80**	**90**	**100**
East Midlands	14.4	16.5	18.7	21.2	23.8
East of England	6.0	8.9	13.0	18.3	24.8
London	1.0	1.4	1.9	2.5	3.3
North East	17.6	18.5	19.5	20.4	21.4
North West	10.9	13.0	15.4	18.0	20.9
South East	6.9	9.7	13.3	17.9	23.5
South West	20.0	22.4	24.9	27.6	30.4
West Midlands	8.2	10.8	13.9	17.6	22.0
Yorkshire and The Humber	12.0	14.1	16.5	19.1	22.1
